# Congenital Cystic Adenomatoid Malformation: Is There a Need for Pregnancy Termination?

**DOI:** 10.1155/2012/397508

**Published:** 2012-03-14

**Authors:** C. Iavazzo, M. Eleftheriades, A. M. Bacanu, D. Hassiakos, D. Botsis

**Affiliations:** 2nd Department of Obstetrics and Gynecology, University of Athens, Aretaieion Hospital, Athens, Greece

## Abstract

*Aim*. Congenital cystic adenomatoid lung malformation is a rare unilateral dysplasia of the lung. Three pathologic types are described in the literature: type I with cysts >2 cm, type II with cysts <1 cm, and type III with microcysts. The aim of this paper is to present a case of a fetus with congenital cystic adenomatoid lung malformation and discuss the necessity for pregnancy termination according to its prognosis and future mortality. *Case*. A 36-year-old pregnant woman (para: 1, gravida: 1) presented in our department for anatomy ultrasound screening at 20 + 1 weeks of gestation. The ultrasound detected a cystic adenomatoid right lung malformation measuring 1.45 × 1.67 cm which caused mediastinal shift of the heart and the lung to the left side. Other findings were cysts of the choroid plexus and echogenic intracardiac foci. The parents after genetic counseling decided pregnancy termination. The pregnant received cabergoline for ablactation. *Conclusion*. Congenital cystic adenomatoid lung malformation has different prognosis according to the type (69% in type I, 0% in types II and III). Fetal hydrops, cardiac and skeletal anomalies, Potter's syndrome, and gastrointestinal atresia are common cofindings. Genetic counseling is necessary, and pregnancy termination is proposed to the cases with poor prognosis.

## 1. Introduction

Congenital cystic adenomatoid lung malformation (CCAM) is a rare, usually, unilateral dysplasia of the lung. The entity was first reported in 1787 with the description of the absence of the lungs [1]. It results from an abnormal maturation of the bronchopulmonary tree, especially an overgrowth of the terminal bronchioles [2]. The etiology of the entity remains unknown. Hamartomatous change in the terminal bronchioles or an arrest in their embryological development between 7 and 15 weeks of gestation is suspected [3]. Decreased apoptosis also plays a role [3]. The related genes in the pathogenesis are HOXB5, Fgf7, and PDGFB [4, 5].

The incidence of CCAM ranges from 1 : 35,000 up to 1 : 25,000 [6]. According to Rosado-de-Christenson and Stocker, three pathologic types are described: type I: cysts diameter >2 cm, type II: cysts diameter <1 cm, and type III: microcysts [7]. In parallel, Adzick et al. differentiated CCAM to macrocystic and microcystic types [8]. The aim of this paper is to present a case of a fetus with congenital cystic adenomatoid lung malformation and discuss the need for pregnancy termination in the cases with poor prognosis.

## 2. Case

**Figure 1:**
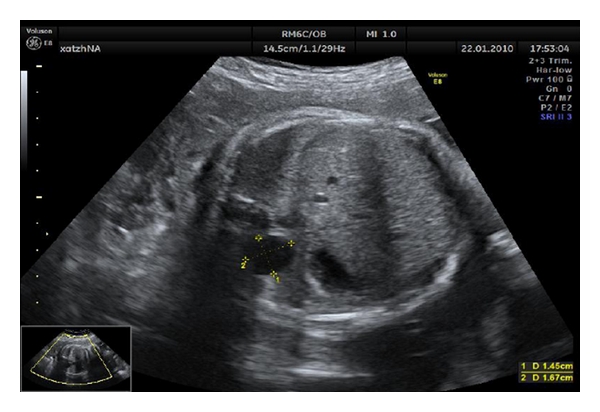
Cystic adenomatoid right lung malformation.

A 36-year-old pregnant woman (para: 1, gravida: 1) presented in our department for anatomy ultrasound screening at 20 + 1 weeks of gestation. No problems are mentioned in her medical or obstetric history. Her current pregnancy was uncomplicated. The ultrasound detected a cystic adenomatoid right lung malformation measuring 1.45 × 1.67 cm which caused mediastinal shift of the heart and the lung to the left side (Figure 1). Other findings were cysts of the choroid plexus, as well as echogenic intracardiac foci. No polyhydramnios or hydrops fetalis were found. The parents after genetic counseling (chromosome analysis) decided pregnancy termination, although they were informed regarding the increased possibility of excellent prognosis. The pregnant received cabergoline for ablactation.

## 3. Discussion

CCAM is a rather rare entity which is usually identified in the anatomy ultrasound scanning of the second trimester. Prenatal diagnosis of CCAM has increased due to the advances in antenatal sonography. The positive predictive value and the sensitivity of 2D sonographic evaluation range between 45–57% and 70–81%, respectively [9]. Moreover, the 3D sonographic evaluation allows all cystic lesions within the studied volume to be seen as opaque or echogenic [10]. MRI is another useful tool for imaging CCAM [11]. In our case, the diagnosis was performed by using 2D ultrasound.

CCAM is usually unilateral [12]. The macrocystic type has large cysts of variable size with thin echogenic areas, whereas the microcystic type has a defined echogenic area with cysts <5 mm in diameter [12]. In our case, a left unilateral anechoic cyst <2 cm was identified. Usually, no association with aneuploidy is found, and for this reason karyotyping is not indicated. Although, in our case, the parents opted for amniocentesis, the karyotype was normal. Some patients especially of type I CCAM demonstrate compression of the heart and the vena cava leading to mediastinal shift, polyhydramnios, and fetal hydrops [13]. Moreover, skeletal anomalies, Potter's syndrome, and gastrointestinal atresia are other common findings [14].

The differential diagnosis includes bronchopulmonary sequestration, bronchogenic cyst, neurenteric cysts, congenital lobar emphysema, and diaphragmatic hernia [11, 15].

The prognosis is usually optimal. Poor prognostic findings are hydrops fetalis, ascites, polyhydramnios, bilateral lung involvement, and a final lung-to-thorax transverse area ratio of less than 0.25 [16, 17]. It should also be mentioned that congenital cystic adenomatoid lung malformation has different prognosis according to the type (69% in type I, 0% in types II and III). The cystic adenomatoid malformation volume ratio—which is defined as the estimated volume of the CCAM divided by head circumference—is used to predict prognosis. A ratio >1.6, leads to a poorer prognosis [18]. In our case, the ratio was less than 1.6 and for this reason termination of pregnancy was not proposed, although the parents opted differently.

Genetic counseling is necessary and pregnancy termination is frequently wrongly proposed due to “poor prognosis.” The good prognosis could be explained by the fact that the cyst decreases in size in utero in the majority of cases [19]. The peak CCAM growth is expected to occur by the 28th gestational week and regression usually occurs in 20% of cases after 29 weeks [19]. Women should be advised to give birth in tertiary centres with a pediatric unit. Antenatally diagnosed CCAM has an excellent prognosis when signs of severe fetal distress are absent. Nakata et al. showed that, in cases with hydrops fetalis, fetal intervention is necessary [6]. Different treatment techniques are proposed in heavier cases such as shunting, laser, and postpartum surgery. Fetal intervention such as cyst-amniotic shunting or tumor resection before 32 weeks of gestation could also be used [20]. Another in utero technique is percutaneous laser ablation [21]. Tran et al. after a retrospective study of 34 cases with CCAM proposed that asymptomatic neonates should have a postnatal CT even if the CCAM appears to have resolved or decreased on antenatal ultrasound [22]. However, in asymptomatic patients elective operation is proposed in 3–6 months of life to avoid respiratory infections, pneumothorax, or even malignancy [23], while a partial lung resection using an axillary skin crease incision is also proposed to obtain a good postoperative quality of life [6]. Such interventions are life saving. Although rare entities, the most common relevant malignancies are bronchoalveolar cancer and rhabdomyosarcoma [24, 25]. Usually, a lobectomy in such cases is performed, and then the mortality rates range from 9 to 49% [26].

In our case, the pregnant woman was advised not to terminate the pregnancy; however, the couple decided to do so because of the increased fear of developing a fetus with hydrops and comorbidity.
